# 1,4,8,11-Tetra­azoniacyclo­tetradecane diaqua­tetra­chloridomanganese(II) dichloride dihydrate

**DOI:** 10.1107/S1600536810031958

**Published:** 2010-08-18

**Authors:** Michaela Pojarová, Karla Fejfarová, Brahim El Bali

**Affiliations:** a Institute of Physics, Na Slovance 2, 182 21 Praha 8, Czech Republic; b Department of Chemistry, Faculty of Sciences, University Mohammed 1st, PO Box 717, 60000 Oujda, Morocco

## Abstract

The title compound, (C_10_H_28_N_4_)[MnCl_4_(H_2_O)_2_]Cl_2_·2H_2_O, consists of isolated octa­hedral [MnCl_4_(H_2_O)_2_]^2−^ anions, tetra­protonated 1,4,8,11-tetra­azoniacyclo­tetradecane cations, chloride anions and water mol­ecules connected by a network of hydrogen bonds. The Mn^II^ atom is situated on an inversion centre, and the 1,4,8,11-tetra­azoniacyclo­tetradecane cation is located on a mirror plane.

## Related literature

For bond distances and angles in the cyclam mol­ecule, see: Melson (1979 ▶).
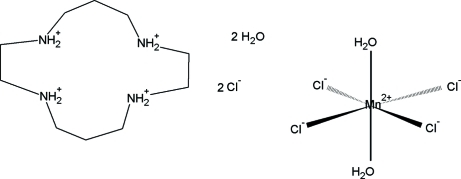


## Experimental

### 

#### Crystal data

(C_10_H_28_N_4_)[MnCl_4_(H_2_O)_2_]Cl_2_·2H_2_O
*M* *_r_* = 544.1
Orthorhombic, 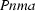

*a* = 14.8492 (2) Å
*b* = 19.3511 (3) Å
*c* = 7.8772 (1) Å
*V* = 2263.50 (5) Å^3^
*Z* = 4
Mo *K*α radiation
μ = 1.31 mm^−1^
*T* = 292 K
0.36 × 0.22 × 0.16 mm

#### Data collection

Oxford Diffraction CCD diffractometer
Absorption correction: analytical (*CrysAlis RED*; Oxford Diffraction, 2005 ▶) *T*_min_ = 0.721, *T*_max_ = 0.840
26200 measured reflections
2429 independent reflections
1999 reflections with *I* > 3σ(*I*)
*R*_int_ = 0.026

#### Refinement

*R*[*F* ^2^ > 2σ(*F* ^2^)] = 0.018
*wR*(*F* ^2^) = 0.059
*S* = 1.09
2429 reflections
133 parameters
H atoms treated by a mixture of independent and constrained refinement
Δρ_max_ = 0.14 e Å^−3^
Δρ_min_ = −0.10 e Å^−3^

### 

Data collection: *CrysAlis CCD* (Oxford Diffraction, 2005 ▶); cell refinement: *CrysAlis RED* (Oxford Diffraction, 2005 ▶); data reduction: *CrysAlis RED*; program(s) used to solve structure: *SIR2002* (Burla *et al.*, 2003 ▶); program(s) used to refine structure: *JANA2006* (Petříček *et al.*, 2006 ▶); molecular graphics: *DIAMOND* (Brandenburg & Putz, 2005 ▶); software used to prepare material for publication: *JANA2006* and *publCIF* (Westrip, 2010 ▶).

## Supplementary Material

Crystal structure: contains datablocks global, I. DOI: 10.1107/S1600536810031958/bt5316sup1.cif

Structure factors: contains datablocks I. DOI: 10.1107/S1600536810031958/bt5316Isup2.hkl

Additional supplementary materials:  crystallographic information; 3D view; checkCIF report

## Figures and Tables

**Table 1:** Hydrogen-bond geometry (Å, °)

*D*—H⋯*A*	*D*—H	H⋯*A*	*D*⋯*A*	*D*—H⋯*A*
N1—H1M⋯O2	0.87	2.01	2.8367 (16)	159
N1—H1N⋯Cl2^i^	0.87	2.51	3.2465 (11)	143
N1—H1N⋯Cl1^ii^	0.87	2.77	3.2317 (11)	115
O1—H1O⋯O2	0.82 (1)	1.94 (1)	2.7474 (15)	172 (2)
O1—H1P⋯Cl3	0.82 (1)	2.35 (1)	3.1382 (10)	162 (1)
N2—H2M⋯O1^iii^	0.87	2.08	2.8926 (15)	155
N2—H2N⋯Cl1	0.87	2.48	3.2383 (11)	146
O2—H2O⋯Cl4^iv^	0.83 (1)	2.19 (1)	3.0205 (12)	173 (1)
O2—H2P⋯Cl2^v^	0.81 (2)	2.52 (2)	3.2832 (11)	157 (1)
C1—H1A⋯Cl2	0.96	2.71	3.6100 (14)	156
C3—H3N⋯Cl3	0.96	2.81	3.7016 (14)	155
C5—H5B⋯Cl4	0.96	2.72	3.6178 (14)	156

Symmetry codes: (i) 
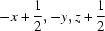
; (ii) 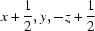; (iii) 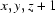; (iv) 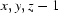; (v) 
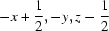
.

**Fig. 1.**
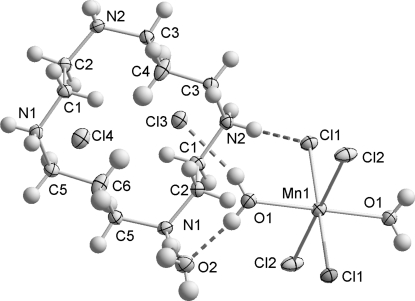
View of the cyclamH44+ cation, the [MnCl4(H2O)2]2- anion, free water molecule and chloride anions, together with atom-labelling scheme. Displacement ellipsoids are shown at the 50% probability level.

**Fig. 2.**
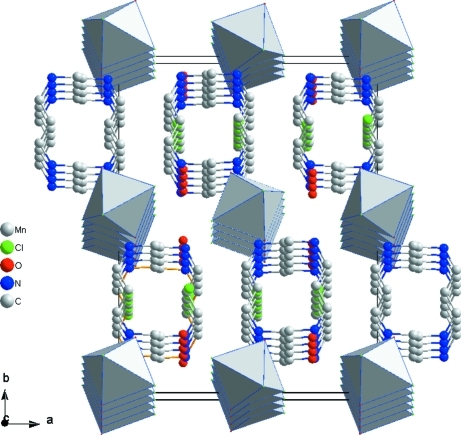
Projection along the c axis, showing the channels formed by the cyclam cavity.

## supplementary crystallographic information

### Comment

The structure contains isolated [MnCl_4_(H_2_O)_2_]^2-^ octahedron and centrosymmetric tetraprotonated 1,4,8,11-tetraazacyclodecane (cyclamH4^4+^) moieties connected by a network of N—H···*X* (*X*= O, Cl) hydrogen bonds (Fig. 1). The positive charge of the cyclamH4^4+^ is out-balanced beside the octahedral anion with two chloride ions. Two molecules of water participate in the manganese coordination, whereas the third water molecule forms bridge between chloride anion (Cl4), nitrogen atom (N1) in 1,4,8,11-tetraazacyclodecane (cyclam) and water (O1) molecule in [MnCl_4_(H_2_O)_2_]. The cyclam molecules and [MnCl4(H_2_O)2] octahedral are arranged into alternating layers parallel with *ab*. In direction of *c* axis the cyclam molecules form infinite channels (Fig. 2). The molecules of cyclam are in a distance of 7.877 Å with chloride anion between the middle CH_2_ groups in propyl chains (C6···Cl4 3.952 Å, Cl4···C6 3.923Å on one side and C4···Cl3 3.855 Å, Cl3···C4 4.022Å on the other side of channels). The tetra-protonated cyclam (C_10_H_28_N_4_)^4+^ exhibits C—C, C—N bond distances and angles in the range usually found for the cyclam molecule (Melson, 1979). The tetra-protonated macrocycle adopts an endodentate quadrangular (3,4,3,4)-A conformation which is the most stable among the four possible conformations, the *exo* orientation of the four nitrogen atoms gives rise to the maximal charge separation. The free water molecule participates also in a cyclic system of hydrogen bonds between water (O1) molecule coordinated to manganese and chloride anions (Cl3 and Cl4). The six-membered cycle is formed by hydrogen bonds between O1—H1o···O2—H2o···Cl4···H2o—O2···H1o—O1—H1p···Cl3···H1p—O1 (Fig. 4).

### Experimental

To an acidic solution of cyclam (1 mmol) was added MnCl_2_.4H_2_O (1 mmol) in 10 ml of distilled water. The mixture was then stirred at room temperature for 3 h after which it was left to evaporate in air. After three weeks, crystals appeared, which were filtered off and washed with 90% ethanol solution.

### Refinement

All hydrogen atoms were discernible in difference Fourier maps and could be refined to reasonable geometry. Despite of it the hydrogen atoms bonded to carbon and nitrogen atoms were constrained to ideal positions. The O—H distances were restrained to 0.82 Å with sigma 0.01. The isotropic temperature parameters of hydrogen atoms were calculated as 1.2**U*_eq_ of the parent atom.)

### Crystal data

**Table:**

(C_10_H_28_N_4_)[MnCl_4_(H_2_O)_2_]Cl_2_·2H_2_O	*F*(000) = 1132
*M**_r_* = 544.1	*D*_x_ = 1.596 Mg m^−^^3^
Orthorhombic, *P**n**m**a*	Mo *K*α radiation, λ = 0.71069 Å
Hall symbol: -P 2ac 2n	Cell parameters from 16479 reflections
*a* = 14.8492 (2) Å	θ = 2.5–26.5°
*b* = 19.3511 (3) Å	µ = 1.31 mm^−^^1^
*c* = 7.8772 (1) Å	*T* = 292 K
*V* = 2263.50 (5) Å^3^	Prism, colourless
*Z* = 4	0.36 × 0.22 × 0.16 mm

### Data collection

**Table:**

Oxford Diffraction CCD diffractometer	2429 independent reflections
Radiation source: X-ray tube	1999 reflections with *I* > 3σ(*I*)
graphite	*R*_int_ = 0.026
Detector resolution: 8.3438 pixels mm^-1^	θ_max_ = 26.5°, θ_min_ = 2.7°
Rotation method data acquisition using ω scans	*h* = −18→18
Absorption correction: analytical (*CrysAlis RED*; Oxford Diffraction, 2005)	*k* = −24→24
*T*_min_ = 0.721, *T*_max_ = 0.840	*l* = −9→9
26200 measured reflections	

### Refinement

**Table:**

Refinement on *F*^2^	Secondary atom site location: difference Fourier map
*R*[*F*^2^ > 2σ(*F*^2^)] = 0.018	Hydrogen site location: difference Fourier map
*wR*(*F*^2^) = 0.059	H atoms treated by a mixture of independent and constrained refinement
*S* = 1.09	Weighting scheme based on measured s.u.'s *w* = 1/[σ^2^(*I*) + 0.0016*I*^2^]
2429 reflections	(Δ/σ)_max_ = 0.010
133 parameters	Δρ_max_ = 0.14 e Å^−^^3^
Primary atom site location: structure-invariant direct methods	Δρ_min_ = −0.10 e Å^−^^3^

### Special details

**Table:**

Experimental. CrysAlis RED, Oxford Diffraction Ltd., Analytical numeric absorption correction using a multifaceted crystal model based on expressions derived by R.C. Clark & J.S. Reid.
Refinement. The refinement was carried out against all reflections. The conventional *R*-factor is always based on *F*. The goodness of fit as well as the weighted *R*-factor are based on *F* and *F*^2^ for refinement carried out on *F* and *F*^2^, respectively. The threshold expression is used only for calculating *R*-factors *etc*. and it is not relevant to the choice of reflections for refinement.All the H atoms were discrenible in difference Fourier maps and could be refined to reasonable geometry. Despite of it the H atoms bonded to catbon and nitrogen atoms were constrained to ideal positions. The O—H distances were restrained to 0.82 Å with σ 0.01. The isotropic temperature parameters of hydrogen atoms were calculated as 1.2**U*_eq_ of the parent atom.The program used for refinement, Jana2006, uses the weighting scheme based on the experimental expectations, see _refine_ls_weighting_details,

### Fractional atomic coordinates and isotropic or equivalent isotropic displacement parameters (Å^2^)

**Table:**

	*x*	*y*	*z*	*U*_iso_*/*U*_eq_	
Mn1	0	0	0	0.01920 (9)	
Cl1	−0.06104 (2)	0.040619 (17)	0.28548 (4)	0.02344 (10)	
Cl3	0.03588 (3)	0.25	0.10590 (6)	0.02742 (14)	
Cl2	0.14001 (2)	−0.046308 (19)	0.14869 (4)	0.03195 (11)	
Cl4	0.27203 (4)	0.25	0.80690 (7)	0.03300 (16)	
O1	0.06526 (7)	0.10208 (5)	−0.04773 (13)	0.0246 (3)	
O2	0.24961 (7)	0.11049 (6)	−0.02640 (14)	0.0323 (3)	
N1	0.25833 (7)	0.12044 (5)	0.33251 (14)	0.0211 (3)	
N2	0.04152 (7)	0.12050 (5)	0.59062 (14)	0.0203 (3)	
C1	0.15939 (9)	0.11683 (7)	0.36796 (16)	0.0212 (4)	
C2	0.14089 (8)	0.11699 (7)	0.55731 (17)	0.0206 (4)	
C3	−0.00618 (8)	0.18592 (7)	0.54150 (17)	0.0217 (4)	
C4	0.03729 (16)	0.25	0.6165 (3)	0.0314 (6)	
C5	0.30646 (9)	0.18591 (6)	0.38037 (17)	0.0228 (4)	
C6	0.26338 (16)	0.25	0.3046 (3)	0.0329 (7)	
H1m	0.268045	0.111373	0.225895	0.0253*	
H1n	0.285152	0.085355	0.379572	0.0253*	
H2m	0.031159	0.111526	0.697062	0.0244*	
H2n	0.015046	0.085424	0.542836	0.0244*	
H1a	0.134992	0.075561	0.318388	0.0254*	
H1b	0.129816	0.155584	0.31619	0.0254*	
H2a	0.165029	0.07569	0.607205	0.0247*	
H2b	0.169981	0.156082	0.608522	0.0247*	
H3a	−0.067928	0.183451	0.577083	0.026*	
H3n	−0.007609	0.18981	0.420039	0.026*	
H4a	0.100476	0.25	0.590704	0.0377*	
H4b	0.029252	0.25	0.737419	0.0377*	
H5a	0.368181	0.18312	0.344749	0.0273*	
H5b	0.308095	0.190111	0.50176	0.0273*	
H6a	0.200057	0.25	0.329167	0.0394*	
H6b	0.272047	0.25	0.183783	0.0394*	
H2o	0.2545 (12)	0.1474 (6)	−0.080 (2)	0.0387*	
H2p	0.2810 (10)	0.0859 (8)	−0.085 (2)	0.0387*	
H1o	0.1201 (6)	0.1002 (8)	−0.042 (2)	0.0296*	
H1p	0.0475 (11)	0.1365 (6)	0.0039 (18)	0.0296*	

### Atomic displacement parameters (Å^2^)

**Table:**

	*U*^11^	*U*^22^	*U*^33^	*U*^12^	*U*^13^	*U*^23^
Mn1	0.02119 (16)	0.01807 (15)	0.01834 (15)	0.00097 (11)	−0.00088 (10)	0.00132 (11)
Cl1	0.0257 (2)	0.02367 (17)	0.02098 (17)	0.00260 (12)	−0.00054 (12)	−0.00266 (13)
Cl3	0.0298 (3)	0.0235 (2)	0.0290 (3)	0	−0.0005 (2)	0
Cl2	0.0316 (2)	0.0361 (2)	0.02823 (19)	0.01481 (15)	−0.00806 (14)	−0.00686 (15)
Cl4	0.0394 (3)	0.0291 (3)	0.0305 (3)	0	−0.0040 (2)	0
O1	0.0262 (5)	0.0217 (5)	0.0260 (5)	−0.0011 (4)	0.0017 (4)	−0.0027 (4)
O2	0.0351 (6)	0.0327 (6)	0.0290 (6)	0.0005 (5)	0.0060 (4)	0.0008 (5)
N1	0.0225 (6)	0.0184 (5)	0.0224 (6)	0.0031 (4)	0.0018 (4)	−0.0012 (5)
N2	0.0223 (6)	0.0179 (5)	0.0207 (6)	−0.0024 (4)	0.0023 (4)	0.0014 (4)
C1	0.0198 (6)	0.0208 (6)	0.0230 (7)	−0.0017 (5)	−0.0010 (5)	−0.0022 (5)
C2	0.0186 (6)	0.0221 (6)	0.0210 (6)	0.0009 (5)	−0.0010 (5)	0.0026 (5)
C3	0.0191 (7)	0.0200 (7)	0.0259 (7)	0.0011 (5)	−0.0014 (5)	0.0002 (6)
C4	0.0448 (13)	0.0191 (9)	0.0304 (11)	0	−0.0135 (10)	0
C5	0.0185 (7)	0.0214 (7)	0.0285 (7)	−0.0010 (5)	−0.0013 (5)	0.0002 (6)
C6	0.0451 (13)	0.0208 (10)	0.0327 (12)	0	−0.0135 (9)	0

### Geometric parameters (Å, °)

**Table:**

Mn1—Cl1	2.5488 (3)	N2—H2n	0.8700
Mn1—Cl1^i^	2.5488 (3)	C1—C2	1.5166 (18)
Mn1—Cl2	2.5490 (4)	C1—H1a	0.9600
Mn1—Cl2^i^	2.5490 (4)	C1—H1b	0.9600
Mn1—O1	2.2322 (10)	C2—H2a	0.9600
Mn1—O1^i^	2.2322 (10)	C2—H2b	0.9600
O1—H1o	0.817 (9)	C3—C4	1.5176 (18)
O1—H1p	0.824 (13)	C3—H3a	0.9600
O2—H2o	0.833 (13)	C3—H3n	0.9600
O2—H2p	0.812 (15)	C4—H4a	0.960
N1—C1	1.4971 (17)	C4—H4b	0.960
N1—C5	1.5026 (16)	C5—C6	1.5179 (18)
N1—H1m	0.8700	C5—H5a	0.9600
N1—H1n	0.8700	C5—H5b	0.9600
N2—C2	1.5004 (16)	C6—H6a	0.960
N2—C3	1.5013 (17)	C6—H6b	0.960
N2—H2m	0.8700		
			
Cl1—Mn1—Cl1^i^	180	C2—C1—H1b	109.47
Cl1—Mn1—Cl2	89.601 (11)	H1a—C1—H1b	107.72
Cl1—Mn1—Cl2^i^	90.399 (11)	N2—C2—C1	110.50 (10)
Cl1—Mn1—O1	91.72 (3)	N2—C2—H2a	109.47
Cl1—Mn1—O1^i^	88.28 (3)	N2—C2—H2b	109.47
Cl1^i^—Mn1—Cl2	90.399 (11)	C1—C2—H2a	109.47
Cl1^i^—Mn1—Cl2^i^	89.601 (11)	C1—C2—H2b	109.47
Cl1^i^—Mn1—O1	88.28 (3)	H2a—C2—H2b	108.42
Cl1^i^—Mn1—O1^i^	91.72 (3)	N2—C3—C4	112.83 (11)
Cl2—Mn1—Cl2^i^	180	N2—C3—H3a	109.47
Cl2—Mn1—O1	91.97 (3)	N2—C3—H3n	109.47
Cl2—Mn1—O1^i^	88.03 (3)	C4—C3—H3a	109.47
Cl2^i^—Mn1—O1	88.03 (3)	C4—C3—H3n	109.47
Cl2^i^—Mn1—O1^i^	91.97 (3)	H3a—C3—H3n	105.89
O1—Mn1—O1^i^	180	C3—C4—C3^ii^	109.58 (15)
H1o—O1—H1p	109.3 (16)	C3—C4—H4a	109.47
H2o—O2—H2p	99.4 (15)	C3—C4—H4b	109.47
C1—N1—C5	117.34 (10)	C3^ii^—C4—H4a	109.47
C1—N1—H1m	109.47	C3^ii^—C4—H4b	109.47
C1—N1—H1n	109.47	H4a—C4—H4b	109.4
C5—N1—H1m	109.47	N1—C5—C6	112.94 (12)
C5—N1—H1n	109.47	N1—C5—H5a	109.47
H1m—N1—H1n	100.26	N1—C5—H5b	109.47
C2—N2—C3	117.20 (10)	C6—C5—H5a	109.47
C2—N2—H2m	109.47	C6—C5—H5b	109.47
C2—N2—H2n	109.47	H5a—C5—H5b	105.77
C3—N2—H2m	109.47	C5—C6—C5^ii^	109.58 (16)
C3—N2—H2n	109.47	C5—C6—H6a	109.47
H2m—N2—H2n	100.45	C5—C6—H6b	109.47
N1—C1—C2	111.17 (10)	C5^ii^—C6—H6a	109.47
N1—C1—H1a	109.47	C5^ii^—C6—H6b	109.47
N1—C1—H1b	109.47	H6a—C6—H6b	109.4
C2—C1—H1a	109.47		

Symmetry codes: (i) −*x*, −*y*, −*z*; (ii) *x*, −*y*+1/2, *z*.

### Hydrogen-bond geometry (Å, °)

**Table:**

*D*—H···*A*	*D*—H	H···*A*	*D*···*A*	*D*—H···*A*
N1—H1M···O2	0.87	2.01	2.8367 (16)	159
N1—H1N···Cl2^iii^	0.87	2.51	3.2465 (11)	143
N1—H1N···Cl1^iv^	0.87	2.77	3.2317 (11)	115
O1—H1O···O2	0.816 (9)	1.937 (9)	2.7474 (15)	171.5 (15)
O1—H1P···Cl3	0.824 (13)	2.345 (12)	3.1382 (10)	161.8 (14)
N2—H2M···O1^v^	0.87	2.08	2.8926 (15)	155
N2—H2N···Cl1	0.87	2.48	3.2383 (11)	146
O2—H2O···Cl4^vi^	0.833 (13)	2.192 (13)	3.0205 (12)	173.4 (14)
O2—H2P···Cl2^vii^	0.810 (15)	2.523 (16)	3.2832 (11)	156.8 (14)
C1—H1A···Cl2	0.96	2.71	3.6100 (14)	156
C3—H3N···Cl3	0.96	2.81	3.7016 (14)	155
C5—H5B···Cl4	0.96	2.72	3.6178 (14)	156

Symmetry codes: (iii) −*x*+1/2, −*y*, *z*+1/2; (iv) *x*+1/2, *y*, −*z*+1/2; (v) *x*, *y*, *z*+1; (vi) *x*, *y*, *z*−1; (vii) −*x*+1/2, −*y*, *z*−1/2.
